# Cascade Skip-Connection BiLSTM Autoencoder for CPR Artifact Removal Prior to AED Shock Advisory

**DOI:** 10.1109/ojcs.2026.3696482

**Published:** 2026-05-25

**Authors:** JAECHAN LIM, DAVID HICKS, MATT VALENTINE, KI H. CHON

**Affiliations:** 1 Department of Biomedical Engineering, University of Connecticut, Storrs, CT 06269 USA; 2 Defibtech LLC, Guilford, CT, 06437 USA

**Keywords:** Automated external defibrillator, BiLSTM autoencoder, cardiopulmonary resuscitation, CPR artifact removal, deep learning, ECG denoising, shock advisory algorithm, ventricular fibrillation

## Abstract

**Background:**

Current automated external defibrillators require pauses in cardiopulmonary resuscitation (CPR) for reliable ECG rhythm analysis, reducing chest compression fraction and delaying defibrillation.

**Methods:**

We propose a skip-connection BiLSTM autoencoder (SBAE) that removes CPR artifacts directly from one-dimensional ECG signals without auxiliary reference signals or time-frequency transformation. A cascade architecture employs a balanced model for initial denoising followed by biased models whose reconstruction is optimized toward one rhythm class. A conservative routing strategy labels cases where the two stages disagree as indeterminate. Performance was evaluated through 10-fold cross-validation with artifact-level data separation using an FDA-approved shock advisory algorithm.

**Results:**

The cascade SBAE achieved 97.51 ± 1.91% sensitivity for ventricular fibrillation, 94.58 ± 3.39% for ventricular tachycardia, 99.30 ± 0.28% specificity for normal sinus rhythm, 98.02 ± 3.02% for asystole, and 97.21 ± 1.05% for other non-shockable rhythms, exceeding American Heart Association thresholds for all categories. The indeterminate rate was 3.63 ± 1.10%.

**Conclusion:**

The proposed framework provides a compact, reference-free solution for shock advisory decision support in automated external defibrillators. Deployment on embedded AED hardware would require additional model optimization and platform-specific engineering.

## I. INTRODUCTION

Sudden cardiac arrest (SCA) remains a leading cause of mortality worldwide. The American Heart Association reports that more than 356,000 out-of-hospital cardiac arrests (OHCA) occur annually in the United States, with approximately 90% resulting in death [1]. The most effective intervention for SCA-induced shockable rhythms, including ventricular fibrillation (VF) and pulseless ventricular tachycardia (VT), is prompt defibrillation combined with high-quality cardiopulmonary resuscitation (CPR) [2]. Automated external defibrillators (AEDs) have become essential components of the chain of survival, enabling bystanders and first responders to deliver life-saving therapy before emergency medical services arrive [3]. However, current AED algorithms require interruption of chest compressions for 8–20 seconds to perform reliable electrocardiogram (ECG) rhythm analysis [4], as mechanical artifacts from CPR obscure the underlying cardiac rhythm.

Minimizing CPR interruptions has a direct impact on survival. Waalewijn et al. [5] demonstrated that survival probability decreases by 10–12% per minute of delayed defibrillation during VF when CPR is not performed. When continuous CPR is maintained, this decline is reduced to 3–4% per minute [6]. Current resuscitation guidelines emphasize maximizing chest compression fraction (CCF), defined as the proportion of resuscitation time during which compressions are delivered [2]. Rhythm analysis pauses directly reduce CCF and delay shock delivery for shockable rhythms, adversely affecting patient outcomes [7].

The fundamental challenge in analyzing ECG during CPR is the severe artifact induced by chest compressions. Compression rates of 100–120 per minute generate quasi-periodic interference concentrated at 1–3 Hz, with harmonics extending into higher frequencies [4]. This spectral content overlaps with diagnostically relevant ECG features, including the QRS complex and fibrillatory waveforms [8]. The resulting signal corruption can cause AED algorithms to misclassify shockable rhythms as non-shockable, delaying necessary defibrillation, or to misclassify non-shockable rhythms as shockable, resulting in inappropriate shock delivery [9].

Various approaches have been proposed for CPR artifact removal and rhythm classification during ongoing compressions. Adaptive filtering methods using reference signals such as compression depth, transthoracic impedance, or accelerometer measurements have demonstrated promising results [4], [8]. However, the majority of commercially available AEDs lack hardware for acquiring these auxiliary signals [10]. Reference-free signal processing methods operating solely on the ECG have been developed [11], [12], but performance degrades when artifact and ECG spectra overlap. Deep learning classifiers can directly output shock decisions from corrupted ECG [13], [14], [15], but these approaches do not provide interpretable denoised signals for subsequent rhythm analysis. Deep learning denoising methods based on convolutional encoder-decoders have shown improved performance [16], [17], though prior approaches employing short-time Fourier transform (STFT) preprocessing introduce computational overhead. The commercial cprINSIGHT system achieves high accuracy when a definitive decision is reached, but requires transthoracic impedance measurements and classifies approximately 30% of cases as indeterminate, necessitating CPR pause [10].

In this paper, we address the problem of ECG rhythm classification during uninterrupted CPR without auxiliary reference signals. We propose a cascade skip-connection BiLSTM autoencoder (SBAE) that removes CPR artifacts directly from one-dimensional ECG signals and applies rhythm-specific refinement through a two-stage classification pipeline that identifies uncertain cases as indeterminate. The anticipated clinical value is more reliable shock/no-shock decisions during ongoing chest compressions, reducing the need for CPR pauses that compromise perfusion.

## II. RELATED WORK

### A. REFERENCE SIGNAL-BASED METHODS

Early approaches to CPR artifact removal relied on auxiliary reference signals correlated with chest compression activity. Kalman filters using compression depth measurements [4], least mean squares (LMS) filters with transthoracic impedance [8], and recursive least squares (RLS) filters [18] have been applied to estimate and subtract the quasi-periodic artifact component. Gabor multipliers have been used for time-frequency localized attenuation of CPR components in VF signals [19]. Gong et al. [20] developed an enhanced adaptive filtering method using acceleration and impedance signals in a porcine model, and a comprehensive review by the same group [21] concluded that reference-based methods generally achieve VF sensitivity above 90% but specificity below the 95% AHA threshold. These methods face a practical barrier: the majority of commercially available AEDs lack hardware for acquiring compression depth, acceleration, or impedance signals.

### B. REFERENCE-FREE SIGNAL PROCESSING

Several algorithms operate solely on the ECG signal. Ruiz de Gauna et al. [11] estimated the fundamental frequency of CPR artifacts from the corrupted ECG and applied notch filtering at harmonic frequencies. Yu et al. [12] combined noise-assisted empirical mode decomposition with LMS filtering, though validation was limited to VF and NSR. Condition-based suppression using spectral characteristics has also been explored [22]. These approaches remain limited by spectral overlap between ECG rhythms and CPR artifacts: when the dominant cardiac frequencies coincide with compression harmonics (typically 1–3 Hz), linear filtering methods distort diagnostically relevant features.

### C. DEEP LEARNING APPROACHES

Deep learning methods for rhythm analysis during CPR fall into two categories: classification-only and denoising.

Classification-only approaches output shock/no-shock decisions without reconstructing the ECG signal. Hajeb-M et al. [13] combined convolutional layers with residual connections and BiLSTM units, achieving 95.2% VF sensitivity and 86.0% specificity. Jekova and Krasteva [14] optimized end-to-end CNNs for OHCA rhythm analysis, reporting approximately 90% VF sensitivity. Isasi et al. [23] used handcrafted features from piston-driven compression recordings. More recently, Krasteva and Jekova [24] compared CNN and CNN-BiLSTM architectures using ECG with optional impedance input, finding that adding impedance improved specificity but that ECG-only models still met AHA sensitivity thresholds. Lee et al. [25] compared multiple neural network structures for identifying shockable rhythm during CPR and demonstrated explainability through gradient-weighted class activation mapping. Isasi et al. [26] extended their earlier work to multiclass rhythm analysis during mechanical CPR, classifying five rhythm categories rather than binary shock/no-shock. While classification-only methods can achieve competitive accuracy, they do not produce denoised signals for post-hoc morphological analysis or integration with existing AED classifier pipelines.

Denoising approaches reconstruct the underlying ECG before classification. Li et al. [17] developed a UNet-based architecture with transfer learning for CPR artifact removal, improving signal-to-noise ratio from −5.3 dB to 1.9 dB. Hajeb-M et al. [27] proposed a deep convolutional encoder-decoder that operated on STFT spectrograms of CPR-corrupted ECG, improving shock advisory accuracy by denoising the two-dimensional time-frequency representation prior to classification. Islam et al. [28] introduced a multi-modal unsupervised approach combining autoencoder-based denoising with signal decomposition, though their evaluation focused on signal quality metrics rather than subsequent classification accuracy. Our prior work [16] employed a cascade convolutional encoder-decoder (CNNED) operating on STFT spectrograms, achieving 95.41% VF sensitivity and 87.66% VT sensitivity with a two-stage refinement strategy. The STFT preprocessing in that approach introduces computational overhead that the present work eliminates by operating directly on one-dimensional ECG.

### D. COMMERCIAL AND CLINICAL SYSTEMS

Two commercial systems for rhythm analysis during chest compressions have been evaluated in clinical settings. The cprINSIGHT algorithm (Stryker LIFEPAK CR2) analyzes ECG and transthoracic impedance signals during ongoing compressions, classifying rhythms as shockable, non-shockable, or requiring pause [10]. In a prospective study of 890 OHCA cases, cprINSIGHT reached a decision during compressions in 70% of analyses, achieving 96% VF sensitivity and 98% NSR specificity for definitive classifications, while 30% required CPR interruption. The Analyze Whilst Compressing (AWC) algorithm was evaluated in the DEFI 2022 study, achieving 91.4–95.4% VF sensitivity and 97.5–100% NSR specificity, with 27.7% of analyses requiring confirmation pauses [29]. Unlike cprINSIGHT, AWC operates on the ECG signal alone without transthoracic impedance input; the impedance traces shown in [29] serve only to visually confirm the presence of chest compressions.

### E. GAPS IN EXISTING WORK

The methods reviewed above have limitations in one or more of the following areas. Reference signal-based methods require auxiliary hardware (accelerometers, impedance sensors) that most commercial AEDs do not provide. Reference-free signal processing methods degrade when cardiac and artifact spectra overlap. Classification-only deep learning methods do not produce denoised signals, preventing morphological verification and limiting integration with existing AED classifiers. Prior denoising approaches that use time-frequency preprocessing (e.g., STFT) add computational cost. Commercial systems that analyze rhythm during CPR achieve high accuracy for definitive decisions but leave 27–30% of cases indeterminate, requiring CPR interruption. No existing ECG-only denoising method has demonstrated performance exceeding all four AHA thresholds (VF sensitivity >90%, VT sensitivity >75%, NSR specificity >99%, other specificity >95%) in a cross-validated evaluation with artifact-level data separation.

## III. MATERIALS AND METHODS

### A. DATASET AND PREPROCESSING

#### 1) TRAINING DATA

Clean ECG recordings for training were obtained from four publicly available PhysioNet databases [30]: the Creighton University Tachyarrhythmia Database (CUDB), the MIT-BIH Malignant Ventricular Arrhythmia Database (VFDB) [31], the Sudden Cardiac Death Holter Database (SDDB), and the MIT-BIH Atrial Fibrillation Database (AFDB). From CUDB, VFDB, and SDDB, all recordings containing non-empty shockable or non-shockable rhythm episodes were used. An additional 21 recordings from AFDB were included to increase the diversity of non-shockable rhythms. Rhythm labels were assigned using the annotation files provided by PhysioNet and verified by visual inspection from trained ECG experts, as described in [16]. Each labeled episode was segmented into 14-second artifact-free samples (1,750 samples at 125 Hz) containing a single continuous rhythm type. Segments with large baseline variations, poor electrode contact, or railed data were excluded based on the annotation files.

Table 1 summarizes the resulting segment counts. In total, 75 unique patients contributed 823 shockable and 23,094 non-shockable segments. Among the 54 patients from CUDB, VFDB, and SDDB, 35 contributed segments to both shockable and non-shockable categories, 10 contributed shockable segments only, and 9 contributed non-shockable segments only. The shockable training segments consist exclusively of ventricular fibrillation (VF); ventricular tachycardia (VT) was not included in the training data. Because the model learns to reconstruct clean ECG waveforms rather than to classify rhythm types, the model must remove artifacts from VT waveforms that were not present during training. The test-set VT sensitivity of 94.58% shows that the autoencoder successfully removes CPR artifacts from VT waveforms despite never seeing VT during training, and the Defibtech algorithm correctly classifies the denoised VT output.

#### 2) TEST DATA

Test ECG recordings were originally compiled by Defibtech, LLC (Guilford, CT) for AED validation [27] and comprise 464 subjects from a separate population not present in any PhysioNet training database: 72 shockable (41 VF, 31 VT) and 392 non-shockable (169 NSR, 33 AFib, 13 AFL, 47 SVT, 21 RSVT, 8 SBC, 9 Asystole, 37 PVC, and 2 Ons - other non-shockable). Each subject contributed a single 14-second recording (1,750 samples at 125 Hz), and the entire recording was used without further segmentation. The complete separation of training and test ECG sources eliminates patient-level data leakage for the clean ECG component.

Some non-shockable rhythm types in the Defibtech test set (e.g., SVT, RSVT, SBC) may not have explicitly labeled counterparts in the PhysioNet training data. This is acceptable because the model is evaluated on its ability to remove CPR artifacts from diverse waveform morphologies, rather than on prior familiarity to each rhythm category, making these cases a direct test of generalization.

#### 3) CPR ARTIFACT DATA AND QUALITY SCREENING

CPR artifact recordings were extracted from Defibtech’s post-market AED log files collected during real out-of-hospital cardiac arrest events, as described in [27]. Recordings of CPR performed during asystole were selected because the negligible ECG amplitude during asystole ensures that the recorded signal consists almost entirely of compression artifact. The recordings were acquired using several Defibtech AED models (primarily the Lifeline DDU-100, the most widely deployed model, along with the DDU-120, DDU-2200, DDU-2300, DDU-2400, and DDU-2450) at a sampling rate of 125 Hz. The AED provides metronome-based coaching for compression rate but does not record compression depth or provide depth feedback; quantitative compression rate and depth statistics are therefore unavailable from the log files. The metronome guides rescuers toward the recommended 100–120 compressions per minute.

The original dataset contained 52 distinct 14-second CPR artifact segments extracted from 43 discrete cardiac arrest events involving 43 different rescuers. The rescuers were predominantly lay users with basic CPR and AED training, as professional paramedics and EMTs typically switch to their own equipment upon arrival and are not captured in public-access AED logs. All recordings were visually confirmed by an experienced cardiology fellow and a trained ECG expert, with a third expert resolving the rare cases of disagreement [27].

Quality screening identified 12 recordings that contained compression pauses exceeding 1 s, visible as near-isoelectric segments within the 14-second window. Because such pauses are inconsistent with the continuous CPR assumption inherent in the synthetic mixing protocol, these 12 recordings were excluded. The remaining 40 recordings, each exhibiting uninterrupted chest compressions throughout the full 14-second duration, were retained for all experiments.

#### 4) CROSS-VALIDATION DESIGN

A 10-fold cross-validation was adopted with the 40 CPR artifact recordings as the partitioning unit. In each fold, 32 artifacts were assigned to training, 4 to validation, and 4 to testing, with no artifact appearing in more than one partition within a fold. This design ensures that the compression waveforms encountered during evaluation are entirely absent from training, so the model is always tested on artifact patterns from rescuers it has never seen.

CPR-contaminated segments were generated by pairing each clean ECG segment with every artifact recording assigned to the corresponding partition at a fixed SNR of −3 dB. Because each ECG segment is combined individually with each artifact, the resulting dataset size scales multiplicatively. For training, the 823 shockable and 23,094 non-shockable clean ECG segments were each paired with all 32 training artifacts, yielding 26,336 (823 × 32) shockable and 739,008 (23,094 × 32) non-shockable contaminated segments per fold. To address the approximately 28:1 class imbalance, shockable training segments were oversampled with replacement to match the non-shockable count, producing 1,478,016 training segments per fold (739,008 for each class). The 4 validation artifacts were paired with all 23,917 clean ECG segments (both shockable and non-shockable), yielding 95,668 (23,917 × 4) validation segments per fold.

Test-set construction followed a different protocol. Only the 464 Defibtech test recordings were used for evaluation, maintaining complete separation from the PhysioNet ECG sources used in training. Each of the 72 shockable and 392 non-shockable test recordings was paired with the 4 held-out test artifacts, producing 288 (72 × 4) shockable and 1,568 (392 × 4) non-shockable test segments per fold. In practice, the actual segment counts may differ slightly from these figures due to discarding of segments that produced NaN or Inf values during z-score normalization.

This design enforces strict independence along two dimensions. No CPR artifact recording is shared between training, validation, and test sets within any fold, and the clean ECG sources for training and testing are drawn from entirely separate patient populations (PhysioNet and Defibtech, respectively). The cross-validation therefore evaluates generalization to both unseen rescuer-specific artifact patterns and unseen patient ECG morphologies simultaneously.

The −3 dB SNR was adopted from our prior work [16] to allow direct comparison with the cascade CNNED baseline. At this level, the CPR artifact power is approximately twice that of the underlying ECG signal. Performance at more severe corruption levels (e.g., −6 dB, −9 dB) may differ and was not evaluated. This constraint is discussed further in the Limitations section.

#### 5) PREPROCESSING

All signals were resampled to 125 Hz. Preprocessing consisted of a third-order Butterworth highpass filter at 0.5 Hz to remove baseline wander, followed by mean removal. Each 14-second segment (1,750 samples) underwent independent z-score normalization:

(1) $$x_{\text{norm}}=\frac{x-\mu }{\sigma },$$

where *μ* and *σ* denote the segment mean and standard deviation, respectively.

#### 6) ETHICAL CONSIDERATIONS

This study utilized de-identified human subject data comprising publicly available ECG recordings from the PhysioNet databases and existing CPR artifact recordings. The original data collectors obtained appropriate ethical approval and patient consent for their respective studies. As this research involved only secondary analysis of anonymized data, it was determined to be exempt from additional institutional review board review.

### B. SBAE ARCHITECTURE

**FIGURE 1.**
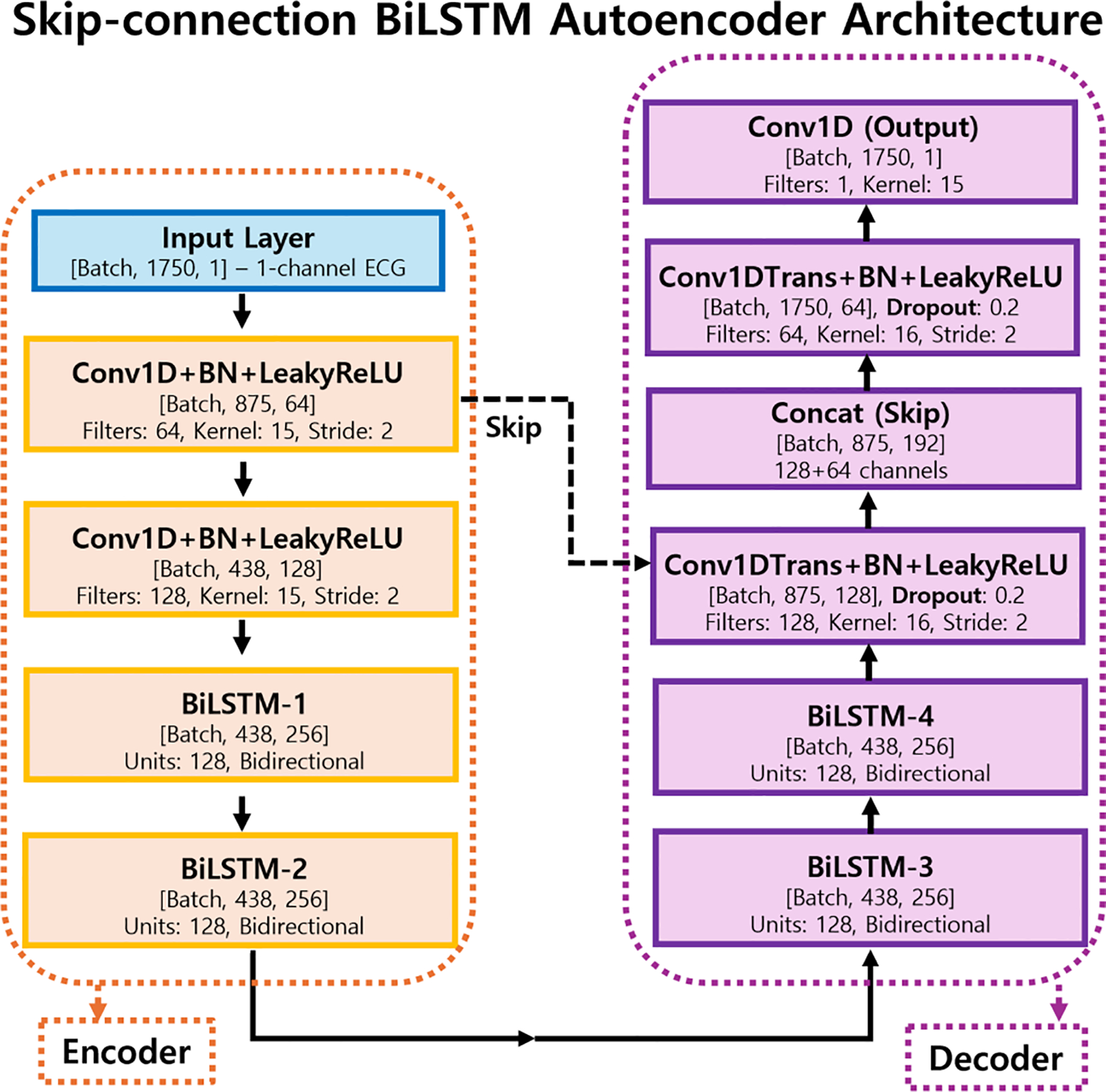
Architecture of the proposed Skip-connection BiLSTM autoencoder (SBAE). The encoder (left) comprises two convolutional layers (kernel size 15) and two bidirectional LSTM layers. The decoder (right) uses transposed convolutions (kernel size 16) for dimensional recovery. A skip connection concatenates early encoder features with decoder features to preserve fine-grained signal characteristics.

We propose a skip-connection BiLSTM autoencoder (SBAE) that operates directly on one-dimensional ECG signals, eliminating the computational overhead of time-frequency transformations such as the short-time Fourier transform employed in prior approaches [16]. The architecture, illustrated in Fig. 1, comprises a convolutional-recurrent encoder and a structurally related but non-symmetric decoder connected by a skip connection.

#### 1) ENCODER

The encoder consists of two convolutional layers followed by two bidirectional LSTM layers. The first convolutional block applies 64 filters with kernel size 15 and stride 2, followed by batch normalization and LeakyReLU activation (negative slope 0.2). The second convolutional block increases the channel dimension to 128 with identical kernel and stride parameters. Odd-sized kernels (15) produce symmetric receptive fields centered on each output sample, which is preferred during feature extraction. The resulting feature maps are transposed and processed by two stacked bidirectional LSTM layers, each with 128 hidden units per direction, producing 256-dimensional outputs.

#### 2) DECODER

The decoder consists of two bidirectional LSTM layers followed by two transposed convolutional layers. The first transposed convolution reduces the channel dimension from 256 to 128 using kernel size 16 and stride 2, with batch normalization, dropout (*p* = 0.2), and LeakyReLU activation. The decoder uses even-sized kernels (16) rather than the encoder’s odd-sized kernels (15) because transposed convolution with stride 2 requires an even kernel size combined with output_padding=1 to produce exact dimensional recovery of the original signal length. A skip connection concatenates the output of the first encoder convolutional layer (64 channels) with the first decoder transposed convolution output (128 channels), yielding 192 channels. The second transposed convolution reduces this to 64 channels. A final convolutional layer with kernel size 15 produces the single-channel denoised output.

Dropout is applied only in the decoder transposed convolution layers. The encoder extracts compressed representations where dropout could discard diagnostically relevant features. The decoder reconstructs full-length signals from these representations, and dropout at this stage serves as a regularizer against overfitting to training-set artifact patterns without degrading the encoder’s feature quality.

Table 2 summarizes the layer specifications and parameter counts.

### C. CASCADE TRAINING STRATEGY

The term “cascade” in this work refers to a multi-model pipeline in which the output of one autoencoder serves as the input to the next, rather than a single model with cascaded internal layers. This two-stage pipeline addresses the limitation that a single model trained on balanced data may not reconstruct both shockable and non-shockable waveforms equally well, leaving residual distortion that affects the subsequent Defibtech classification differently for each rhythm class. The biased models compensate for this by specializing their reconstruction toward one rhythm class, reducing the class-specific residual distortion that the balanced model leaves behind. For example, if the balanced model correctly classifies a segment as shockable, the shockable-biased model further clarifies the shockable waveform morphology, reinforcing the initial classification. However, if the segment is actually non-shockable but was misclassified as shockable, the shockable-biased model concentrates its reconstruction on shockable features that are not inherent in the underlying signal, allowing non-shockable characteristics to emerge more prominently in the output and potentially producing a non-shockable classification in the second stage.

#### 1) STAGE 1: BALANCED MODEL

The first-stage SBAE is trained on balanced data (50% shockable, 50% non-shockable) to provide general-purpose CPR artifact removal. Training employs the Adam optimizer with an initial learning rate of 0.001, reduced by a factor of 0.1 every 4 epochs. The batch size is 64, and training proceeds for a maximum of 20 epochs with early stopping (patience of 5 epochs) based on validation loss. Gradient clipping with maximum norm 1.0 prevents gradient explosion. The loss function is mean squared error (MSE) between the denoised output and clean ECG target:

(2) $${ℒ}_{\text{MSE}}=\frac{1}{N}\overset{N}{\underset{i=1}{\sum }}\|{\hat{x}}_{i}-x_{i}{\|}^{2},$$

where ${\hat{x}}_{i}$ and *x*_*i*_ denote the denoised and clean ECG segments, respectively.

#### 2) STAGE 2: BIASED MODELS

Two rhythm-specific models are trained on the outputs of the balanced model. The shockable-biased model (denoted 20nsh) is trained on data comprising 80% shockable and 20% non-shockable samples. Conversely, the non-shockable-biased model (denoted 20sh) is trained on 80% non-shockable and 20% shockable samples. The naming convention denotes the minority class proportion rather than the bias direction. Because each biased model sees one rhythm class four times more frequently during training, its reconstruction is optimized toward recovering that class’s waveform morphology. The inputs to these biased models are the denoised outputs from the balanced model, while the targets remain the original clean ECG signals. Each biased model therefore learns to reduce the class-specific residual distortion that the balanced model’s equal weighting of both classes leaves behind. Both biased models use the same MSE loss function (ℒ MSE) and identical training hyperparameters as the balanced model. The 80/20 split ratio was selected heuristically based on our prior work [16]; a systematic grid search over split ratios was not performed and remains a direction for future work.

Table 3 provides the complete training configuration and hardware specifications.

### D. CASCADE ROUTING LOGIC

During inference, the cascade system uses the Defibtech shock advisory algorithm at two decision points to route each segment through the appropriate biased model and to determine whether the final classification is reliable. The routing procedure operates as follows:

1. The CPR-contaminated ECG segment is processed through the balanced SBAE model.
2. The denoised output is classified by the Defibtech algorithm as shockable or non-shockable (Classification 1).
3. Based on Classification 1, the denoised output is passed to the corresponding biased model for further reconstruction:
  - If classified as shockable, the denoised output is processed by the 20nsh model (shockable-biased).
  - If classified as non-shockable, the denoised output is processed by the 20sh model (non-shockable-biased).
4. The biased model’s output is reclassified by the Defibtech algorithm (Classification 2).
5. If Classification 1 and Classification 2 agree, the decision is finalized. If they disagree, the segment is labeled indeterminate.

Fig. 2

adapted from [16] illustrates this routing procedure. Because the biased model is trained to reinforce reconstruction of the rhythm class indicated by Classification 1, disagreement between the two stages indicates that the segment cannot be reliably classified. In clinical deployment, an indeterminate result would prompt reanalysis of the next available ECG segment, or if successive segments remain indeterminate, a brief CPR pause for clean signal acquisition [16].

### E. EVALUATION PROTOCOL

Performance was evaluated using the Defibtech shock advisory algorithm, an FDA-approved AED classifier (PMA P160032) [32], as the ground-truth decision system. Sensitivity (SE) was computed for shockable rhythms (VF, VT) and specificity (SP) for non-shockable rhythms (NSR, others):

(3) $$\text{SE}=\frac{\text{TP}}{\text{TP}+\text{FN}},\;\text{SP}=\frac{\text{TN}}{\text{TN}+\text{FP}},$$

where TP, TN, FP, and FN denote true positives, true negatives, false positives, and false negatives, respectively.

For cascade models, indeterminate cases (where Classification 1 and Classification 2 disagree) were excluded from SE/SP calculations, following the convention in [16]. The percentage of indeterminate cases was reported separately for each rhythm type. Performance thresholds established by the American Heart Association (AHA) [33] require SE > 90% for coarse VF, SE > 75% for rapid VT, SP > 99% for NSR, and SP > 95% for other non-shockable rhythms.

Inference time was measured on a 24-core AMD EPYC 7402P CPU at 2.79 GHz to provide a computational benchmark. All timing measurements were conducted on 14-second segments.

## IV. RESULTS

### A. DEFIBTECH ALGORITHM BASELINE PERFORMANCE

To establish the reliability of the Defibtech shock advisory algorithm as an evaluation tool, we first assessed its performance on the 464 clean ECG test segments without CPR artifacts (Table 4). The algorithm achieved 100% sensitivity for VF and 96.77% for VT (1 of 31 segments misclassified), with 100% specificity for NSR, Asystole, and all other non-shockable rhythm types except SVT, where 2 of 47 segments were misclassified as shockable. Overall sensitivity was 98.61% and specificity was 99.49%.

### B. SHOCK ADVISORY PERFORMANCE

Table 5 presents shock advisory performance across four configurations evaluated through 10-fold cross-validation. The unfiltered CPR-corrupted ECG baseline shows severe degradation: VF sensitivity dropped to 71.95 ± 6.18% and VT sensitivity to 68.55 ± 7.53%, both well below AHA thresholds. Asystole specificity is reported separately given its clinical importance as the most frequent rhythm in out-of-hospital cardiac arrest.

The balanced model alone recovered most of the performance lost to CPR artifacts but did not meet the AHA threshold for NSR specificity (98.82% vs. required >99%). Adding the cascade architecture with an override strategy, where the biased model decision is always used as the final classification, yielded minimal additional gain (e.g., VF sensitivity 94.63% vs. 94.09%). This indicates that biased models introduce roughly as many new errors as they correct.

The conservative routing strategy, where disagreements between models are labeled indeterminate, produced the best results across all metrics. VF sensitivity reached 97.51 ± 1.91%, VT sensitivity 94.58 ± 3.39%, NSR specificity 99.30 ± 0.28%, Asystole specificity 98.02 ± 3.02%, and other non-shockable specificity 97.21 ± 1.05%. All metrics exceed AHA thresholds [33]. The improvement over the override strategy confirms that the cascade architecture’s primary value lies in uncertainty detection rather than decision correction.

### C. INDETERMINATE CASE ANALYSIS

Table 6 shows the distribution of indeterminate cases by rhythm type for the conservative routing strategy. The overall indeterminate rate was 3.63 ± 1.10%, ranging from 2.64% to 5.98% across folds.

Indeterminate rates were higher for shockable rhythms (VF: 6.65%, VT: 11.29%) than non-shockable rhythms (NSR: 1.20%, Asystole: 2.50%). This reflects the greater difficulty of denoising shockable rhythms at −3 dB, where CPR artifact energy can overlap with the chaotic frequency content of VF and the rapid oscillations of VT. Among non-shockable rhythms, AFib had the highest indeterminate rate (6.52%), consistent with the irregular rhythm pattern being more susceptible to misclassification as shockable when combined with residual CPR artifacts.

### D. COMPUTATIONAL ANALYSIS

Table 7 summarizes model complexity and inference time. The single-stage SBAE contains 2,296,833 parameters (4.38 MB at FP16 precision), while the cascade configuration doubles this to 4,593,666 parameters (8.76 MB). Inference time for 14-second segments averaged 55.10 ms for SBAE and 112.13 ms for cascade SBAE on a server-class CPU (AMD EPYC 7402P, Python/PyTorch). These measurements serve as computational benchmarks; deployment on embedded AED hardware would require additional optimization including model compression, quantization, and platform-specific engineering.

### E. VISUAL EXAMPLES

**FIGURE 3.**
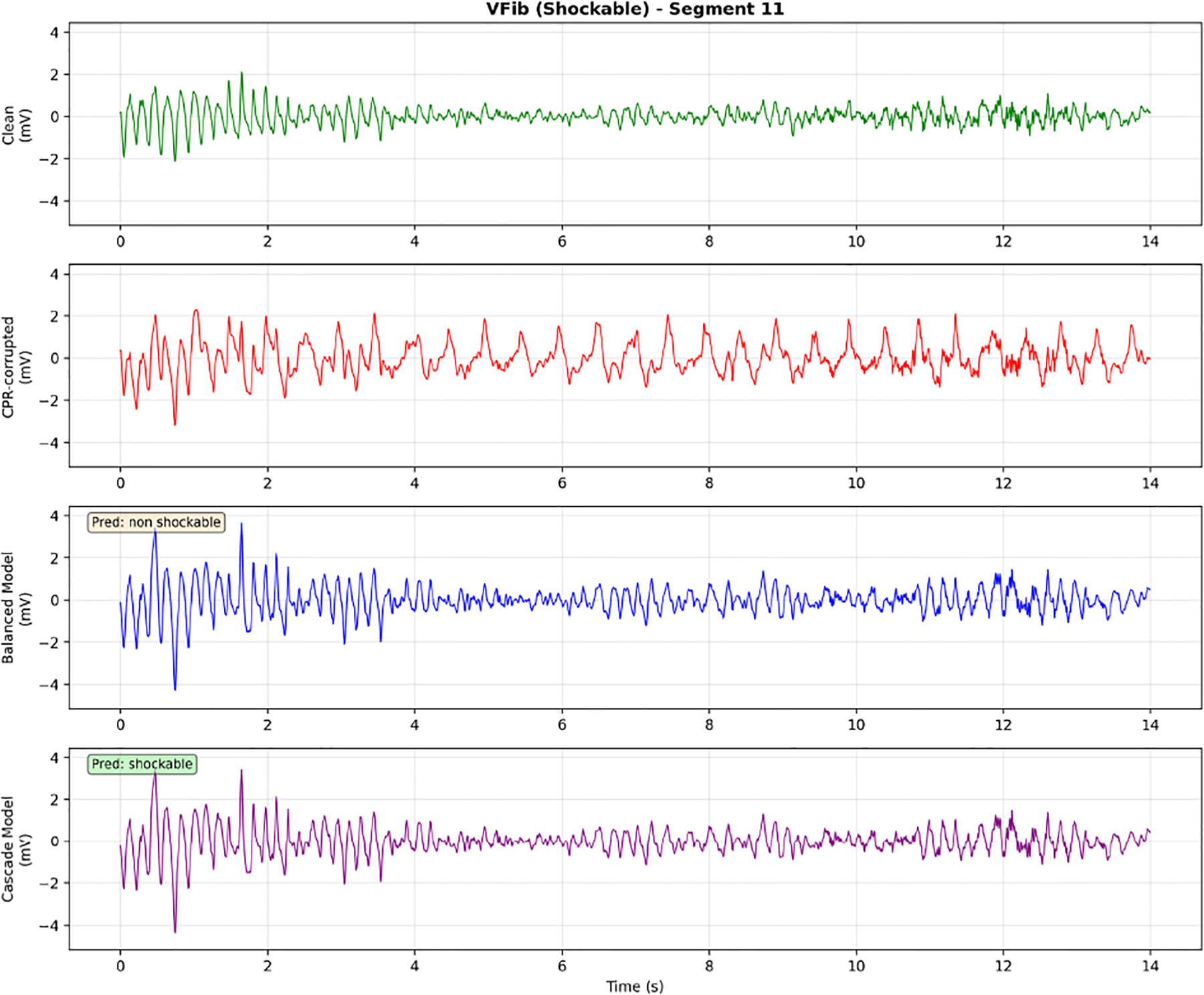
Indeterminate case for VF (shockable). From top to bottom: clean ECG, CPR-corrupted signal, balanced model output, and cascade model output. The balanced model output is classified as non-shockable by the Defibtech algorithm, while the cascade model output is correctly classified as shockable.

**FIGURE 4.**
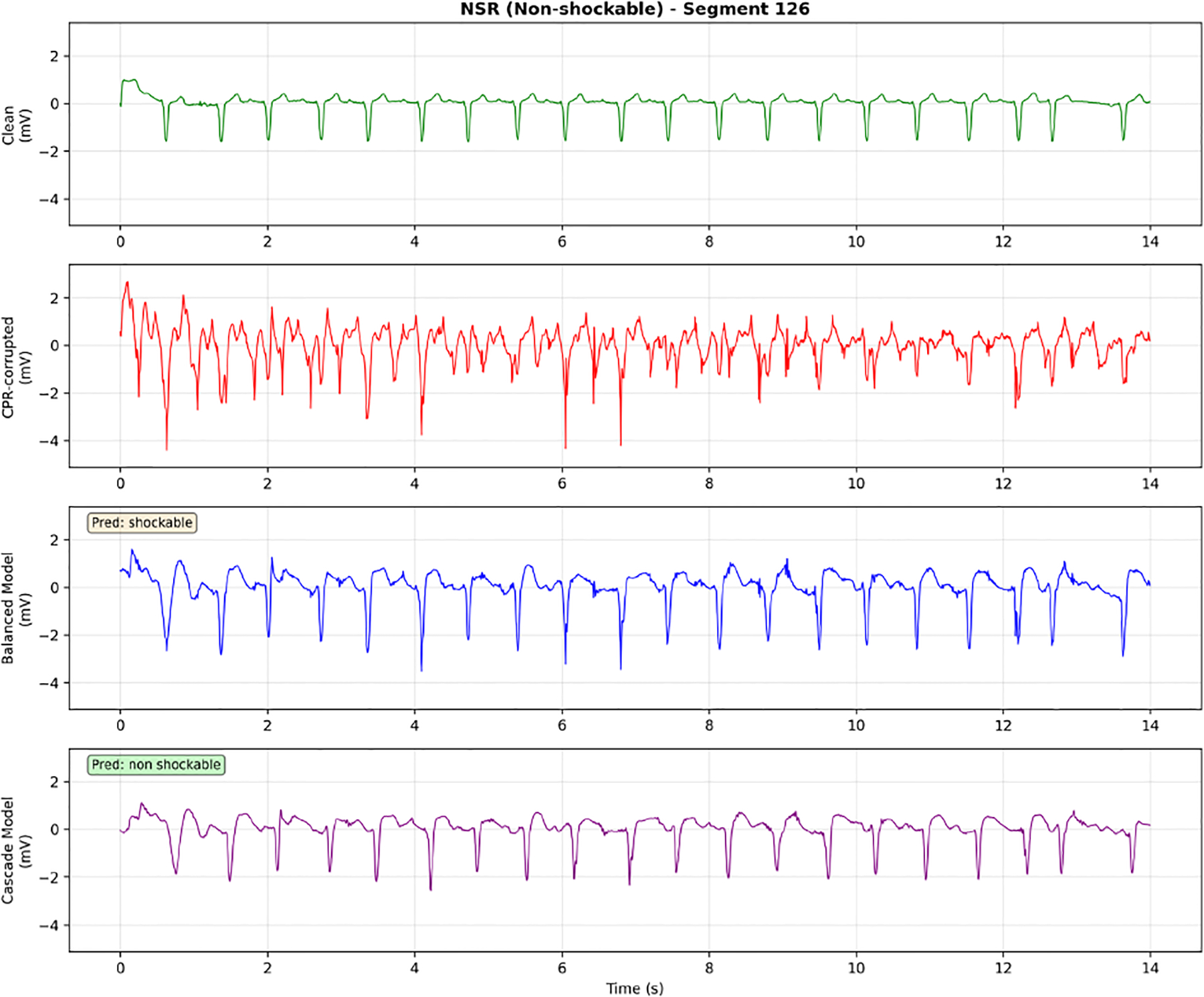
Indeterminate case for NSR (non-shockable). From top to bottom: clean ECG, CPR-corrupted signal, balanced model output, and cascade model output. The balanced model output is classified as shockable by the Defibtech algorithm, while the cascade model output is correctly classified as non-shockable. The cascade refinement attenuated residual CPR artifacts while preserving regular QRS periodicity.

Figs. 3 and 4 show representative indeterminate cases where the balanced and cascade models produced different classifications. Each figure displays four traces: clean ECG (top), CPR-corrupted signal, balanced model output with Defibtech classification, and cascade model output with Defibtech classification.

For the VF example (Fig. 3), the balanced model output was classified as non-shockable due to residual artifacts that suppressed the fibrillatory waveform. The cascade refinement using the 20sh model (triggered by the initial non-shockable classification) recovered the VF morphology, and the cascade output was correctly classified as shockable. Because Classification 1 (non-shockable) and Classification 2 (shockable) disagree, this segment is labeled indeterminate under the conservative strategy, illustrating how the cascade identifies cases where the balanced model’s initial classification is unreliable.

For the NSR example (Fig. 4), the balanced model output was classified as shockable, likely due to residual CPR artifacts in frequency ranges used by the Defibtech algorithm for rhythm discrimination. The cascade refinement using the 20nsh model attenuated residual CPR artifacts while preserving regular QRS periodicity, and the cascade output was correctly classified as non-shockable. As shown in Table 4, the Defibtech algorithm achieves 100% specificity for NSR on clean signals, confirming that the misclassification is attributable to residual artifact rather than classifier instability. Because Classification 1 (shockable) and Classification 2 (non-shockable) disagree, this segment is also labeled indeterminate, preventing an incorrect shock recommendation.

## V. DISCUSSION

### A. CASCADE ROUTING: OVERRIDE VS. CONSERVATIVE

The ablation results in Table 5 show that the cascade architecture’s value lies in uncertainty detection rather than decision correction. The override strategy, where the biased model always determines the final classification, improved VF sensitivity by only 0.5 percentage points over the balanced model alone (94.63% vs. 94.09%). The conservative strategy, where disagreements are labeled indeterminate, raised VF sensitivity to 97.51% and VT sensitivity to 94.58%. The near-identical performance of the override and balanced-only configurations indicates that, within the disagreement set, the biased model is not systematically more accurate than the balanced model. Overriding with the biased model’s decision therefore does not produce a net reduction in classification errors. Excluding these disagreement cases from the final output, as the conservative strategy does, yields the observed performance gains.

The effectiveness of the conservative strategy follows from how the biased models are constructed. When the balanced model classifies a segment as shockable, the segment is routed to the 20nsh model, which was trained on 80% shockable and 20% non-shockable data. Because this model saw shockable patterns four times more frequently during training, its learned reconstruction is strongly biased toward recovering shockable waveform morphologies, and the Defibtech classifier applied to this output is therefore more likely to confirm a shockable classification. If the 20nsh model instead produces a non-shockable classification, the segment contains features strong enough to produce a non-shockable classification even after reconstruction by a model whose denoising was shaped predominantly by shockable training examples. The same logic applies in reverse: a non-shockable classification is routed to the 20sh model (80% non-shockable), whose reconstruction favors non-shockable morphologies. Disagreement under these conditions carries more weight than disagreement between two unbiased models would, because the second model was deliberately trained to reinforce the first-stage decision. As a result, the disagreement set is enriched with segments that were misclassified in the first stage. Removing 3.63% of segments as indeterminate raised VT sensitivity from 89.35% to 94.58%, indicating that the disagreement set contains a substantially higher concentration of misclassified segments than the agreement set.

This conservative design has a clinical trade-off. The 3.63% indeterminate rate means that for roughly 1 in 27 analysis windows, the system cannot provide a shock/no-shock decision. In practice, an indeterminate result during CPR would trigger reanalysis of the next available segment. If successive segments also return indeterminate, a brief CPR pause for clean ECG acquisition may still be needed. The indeterminate rate varies by rhythm: VT segments were indeterminate 11.29% of the time, compared to 1.20% for NSR. This asymmetry reflects the greater denoising difficulty for VT, where rapid periodic oscillations at 150–250 bpm can be partially masked by CPR artifact energy in overlapping frequency bands. Prior work comparing AED analysis strategies has similarly highlighted the trade-off between decision coverage and decision reliability [34].

### B. COMPARISON WITH PRIOR WORK

Table 8 compares the proposed method with prior work. Direct comparison across studies is difficult because test datasets, rhythm compositions, corruption protocols, and evaluation metrics differ. We therefore restrict our discussion to two specific comparisons where conditions are more closely matched.

First, the cascade SBAE can be compared directly with our prior cascade CNNED [16], since both use the same Defibtech test ECG segments and CPR artifact recordings at −3 dB SNR. The SBAE significantly improved VT sensitivity to 94.58% (from 87.66%) while also improving VF sensitivity (97.51% vs. 95.41%) and maintaining equivalent NSR specificity (99.30% vs. 99.35%). The improvement for VT is consistent with the BiLSTM encoder’s ability to capture temporal dependencies in the rapid periodic VT waveform, though this comparison also involves other design changes beyond the architecture type, as noted in the Limitations section. The SBAE also eliminates the STFT preprocessing step required by the CNNED’s two-dimensional spectrogram input, reducing computational complexity.

Second, the commercial cprINSIGHT [10] and AWC [29] systems were evaluated on real OHCA recordings with heterogeneous artifact conditions, not at a fixed −3 dB SNR. The 30% and 27.7% indeterminate rates reported for these systems therefore cannot be directly compared with our 3.63% rate. The clinical recordings contain a wider range of artifact intensities, rhythm morphologies, and noise sources than our synthetic mixing protocol. Additionally, cprINSIGHT uses transthoracic impedance as an auxiliary input, while AWC operates on ECG alone [29]. We acknowledge that our indeterminate rate would likely increase on real OHCA data with more variable artifact conditions.

### C. LIMITATIONS

The following limitations should be considered when interpreting these results.

The CPR artifact dataset contained 40 recordings from 40 rescuers after quality screening. This sample may not capture the full variability of compression styles encountered in OHCA. Rescuer-specific differences in compression depth, rate, duty cycle, and hand positioning produce artifact patterns that could differ from those in our dataset. Expanding the artifact database with recordings from a larger and more diverse rescuer population would strengthen generalizability.

The test data were generated by synthetically combining clean ECG with CPR artifacts at a fixed SNR of −3 dB. This protocol enables controlled evaluation with known ground truth but does not replicate the complex electromechanical interactions present in real OHCA recordings, where artifact amplitude varies within a single episode and additional noise sources (motion, electrode contact, EMG) are present. Performance at lower SNR values (e.g., −6 dB, −9 dB) may differ and was not evaluated. Validation on real OHCA recordings is needed to establish clinical applicability.

The number of test segments per rhythm type is uneven. Although 9 non-shockable rhythm types are included, several categories contain few segments (e.g., SBC: 8 segments, Asystole: 9 segments), which contributes to higher variance in fold-level performance for these rhythms. The shockable class is also restricted to VF and VT; polymorphic VT and pulseless electrical activity were not included.

The architecture uses four BiLSTM layers totaling 4.6 million parameters. While inference time on a server-class CPU (112 ms for 14 seconds of ECG) is well within the analysis window, this measurement does not reflect performance on embedded AED hardware. Deployment would require model compression, quantization, and platform-specific optimization, as demonstrated for simpler architectures in [35], [36]. A systematic comparison with a purely convolutional alternative of matched capacity was not performed; we justified the BiLSTM choice by the VT sensitivity improvement over the convolutional CNNED baseline [16] (94.58% vs. 87.66%), but acknowledge that this comparison confounds architecture type with other design changes.

The 80/20 class ratio for biased model training was chosen heuristically from [16]. A systematic grid search over split ratios was not conducted and could identify a more effective ratio.

Signal reconstruction quality was evaluated only through the downstream shock advisory outcome, not through signal-level metrics such as root mean square error or correlation between the denoised and original clean ECG. While Figs. 3 and 4 provide qualitative evidence that the model preserves rhythm-specific waveform morphology, a systematic quantitative assessment of reconstruction fidelity across all rhythm types and folds was not performed and could be addressed in future work.

## VI. CONCLUSION

We proposed a cascade skip-connection BiLSTM autoencoder for removing CPR artifacts from ECG signals during chest compressions. The system operates on one-dimensional ECG without auxiliary reference signals or time-frequency preprocessing. A balanced autoencoder provides initial denoising, and biased models, each with reconstruction tailored toward one rhythm class, refine the output based on the initial classification. When the two classification stages disagree, the segment is labeled indeterminate. Most misclassifications fall within this disagreement set, so removing these cases from the final output improved classification accuracy.

The cascade SBAE exceeded AHA thresholds for all rhythm categories under 10-fold cross-validation with strict artifact-level separation. The conservative routing strategy proved more effective than an override strategy, indicating that the cascade architecture’s primary contribution is identifying uncertain cases rather than correcting misclassifications, an important safety property for AED deployment.

Validation on real OHCA recordings, expansion of the artifact database, evaluation at multiple SNR levels, and deployment optimization for embedded AED hardware are needed before clinical translation.

## Figures and Tables

**FIGURE 2.**
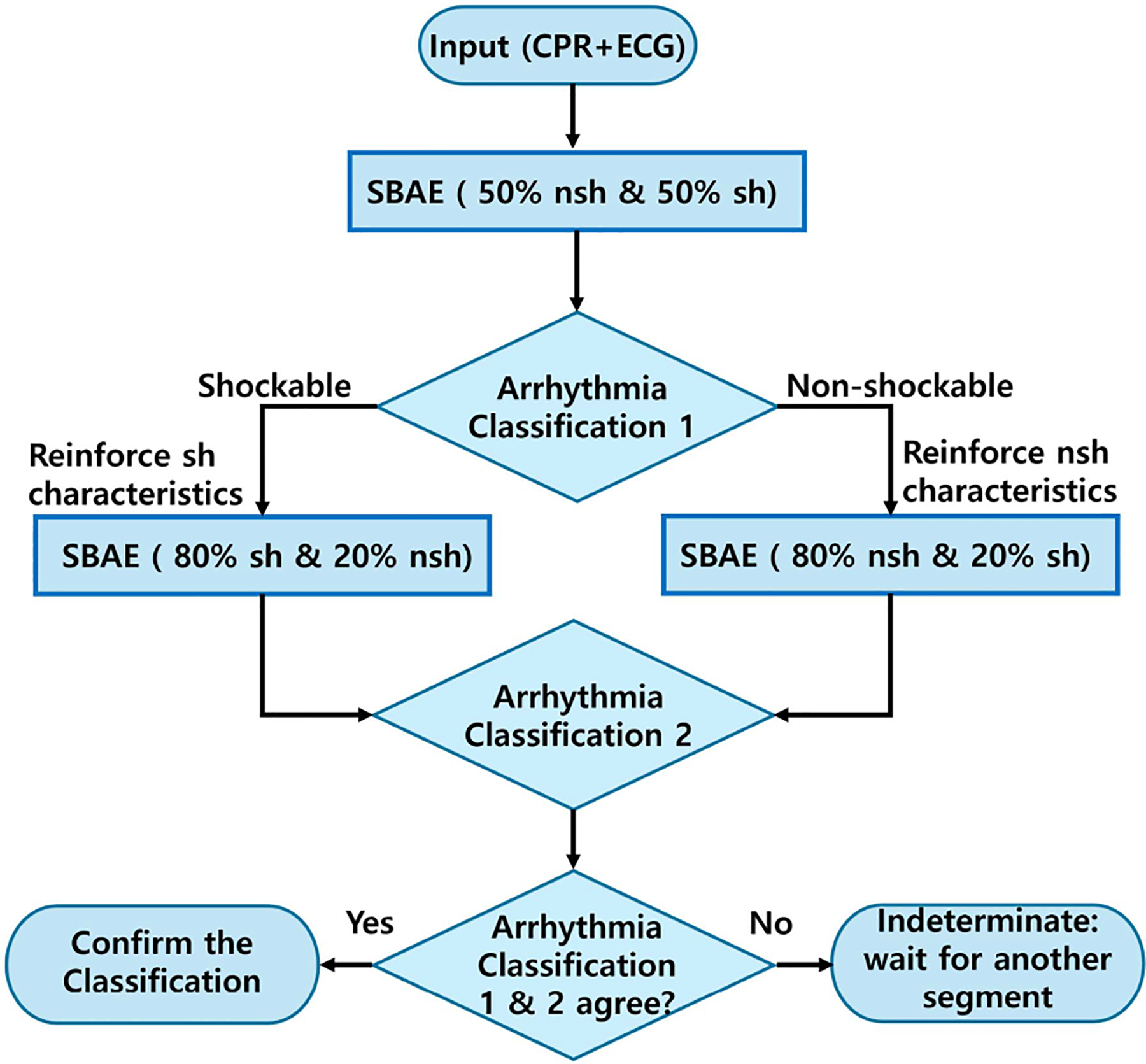
Flowchart of the cascade routing logic adapted from [16]. The CPR-contaminated ECG is first processed by the balanced SBAE model and classified (Classification 1). Based on this result, the signal is routed to the appropriate biased model for refinement and reclassified (Classification 2). Agreement between the two classifications confirms the decision; disagreement yields an indeterminate outcome.

**TABLE 1.** Training Data: Clean ECG Segments by Database and Rhythm Type

Database	Rhythm type	Patients	Segments
CUDB	VF (shockable)	21	165
VFDB	VF (shockable)	8	317
SDDB	VF (shockable)	15	341
Shockable subtotal		44*	823
CUDB	NSR/Other (non-shockable)	18	477
VFDB	NSR/Other (non-shockable)	5	537
SDDB	NSR/Other (non-shockable)	21	2,712
AFDB	AFib (non-shockable)	21	19,368
Non-shockable subtotal		65*	23,094
**Total unique patients**		**75**	**23,917**

* Some patients contributed segments to both SH/NSH categories; column sums exceed the unique patient total.

**TABLE 2.** Architecture Specifications of the Proposed SBAE Model. *B* Denotes Batch Size

Component	Layer Type	Kernel/Stride	Parameters	Output Shape
*Encoder*				
Conv1D-1	Conv1D + BatchNorm + LeakyReLU	15/2	1,024	(*B*, 64, 875)
Conv1D-2	Conv1D + BatchNorm + LeakyReLU	15/2	123,008	(*B,* 128, 438)
BiLSTM-1	Bidirectional LSTM	—	263,168	(*B,* 438, 256)
BiLSTM-2	Bidirectional LSTM	—	395,264	(*B,* 438, 256)
*Decoder*				
BiLSTM-1	Bidirectional LSTM	—	395,264	(*B*, 438, 256)
BiLSTM-2	Bidirectional LSTM	—	395,264	(*B,* 438, 256)
ConvT1D-1	ConvTranspose1D + Dropout	16/2	524,416	(*B,* 128, 875)
Skip Connection	Concatenate with Conv1D-1	—	—	(*B*, 192, 875)
ConvT1D-2	ConvTranspose1D + Dropout	16/2	196,672	(*B,* 64, 1750)
Output Conv	Conv1D	15/1	961	(*B,* 1, 1750)
Total (SBAE)			2,296,833	—

**TABLE 3.** Training Hyperparameters and Implementation Details

Hyperparameter	Value
*Data Configuration*	
Input length	1,750 samples (14 s at 125 Hz)
Normalization	Per-segment z-score
SNR (artifact addition)	−3 dB
*Training Configuration*	
Loss function	Mean Squared Error (MSE)
Optimizer	Adam
Learning rate	0.001
Batch size	64
Maximum epochs	20
Early stopping patience	5 epochs (observed stop: epochs 2–6)
Gradient clipping	max_norm = 1.0
Dropout rate	0.2 (decoder ConvTranspose layers only)
LeakyReLU negative slope	0.2
*Cascade Training*	
Balanced model	50% shockable, 50% non-shockable
Biased model (20sh)	20% shockable, 80% non-shockable
Biased model (20nsh)	80% shockable, 20% non-shockable
*Hardware*	
Training	NVIDIA L40 GPU
Inference benchmark	AMD EPYC 7402P CPU (24-core, 2.79 GHz)

**TABLE 4.** Baseline Performance of the Defibtech Shock Advisory Algorithm on Clean ECG (Without CPR Artifacts)

Rhythm	Type	Segments	Correct	Performance
VF	Shockable	41	41	SE: 100.00%
VT	Shockable	31	30	SE: 96.77%
NSR	Non-shockable	169	169	SP: 100.00%
Asystole	Non-shockable	9	9	SP: 100.00%
Other^a^	Non-shockable	214	212	SP: 99.07%
**Overall**			**461/464**	SE: 98.61%, SP: 99.49%

a Includes AFib, AFL, SVT, RSVT, SBC, PVC, and Ons. Two SVT segments were misclassified as shockable.

**TABLE 5.** Defibtech Shock Advisory Performance on CPR-Contaminated ECG Before and After Denoising (10-Fold Cross-Validation, Mean ± Std [%])

Configuration	VF (SE)	VT (SE)	NSR (SP)	Asys (SP)	Other (SP)^a^
AHA Threshold	>90	>75	>99	>95	>95
Unfiltered ECG	71.95±6.18	68.55±7.53	96.57±1.42	93.33±2.68	91.66±2.41
Balanced only	94.09±3.82	89.35±3.68	98.82±0.59	96.39±2.64	95.24±1.54
Cascade (override)	94.63±1.92	89.68±3.87	98.59±0.67	97.22±3.46	95.55±1.54
Cascade (conservative)	**97.51±1.91**	**94.58±3.39**	**99.30±0.28**	**98.02±3.02**	**97.21±1.05**

a Includes all non-shockable rhythms other than NSR and Asystole: AFib, AFL,SVT, RSVT, SBC, PVC, and Ons.

**TABLE 6.** Indeterminate Rate by Rhythm Type (10-Fold Cross-Validation, Mean ± Std)

Rhythm	Samples/fold	Indeterminate Rate
*Shockable*		
VF	164	6.65 ± 2.81%
VT	124	11.29 ± 3.36%
*Non-shockable*		
NSR	676	1.20 ± 0.72%
AFib	132	6.52 ± 1.96%
Ons^a^	220	3.05 ± 1.73%
Asystole	36	2.50 ± 2.05%
AFL	52	3.46 ± 1.77%
SVT	188	4.26 ± 1.83%
RSVT	84	0.60 ± 0.84%
SBC	32	4.38 ± 4.70%
PVC	148	4.39 ± 3.96%
**Overall**	**1,856**	**3.63 ± 1.10%**

a Other non-shockable rhythms as categorized in the Defibtech test dataset.

**TABLE 7.** Model Complexity and Inference Time

Model	Parameters	Size (MB)	Time (ms)^a^
SBAE	2,296,833	4.38	55.10±0.38
Cascade SBAE	4,593,666	8.76	112.13±0.67

a Measured on AMD EPYC 7402P CPU using Python/PyTorch. Not directly representative of embedded AED hardware performance.

**TABLE 8.** Comparison of CPR Artifact Removal and Rhythm Classification Methods

Study	Year	Test Data	Reference	Method	SE VF^a^	SE VT^a^	SP NSR^a^	SP Other^a^
Ruiz de Gauna [11]	2008	Real OHCA	None	Kalman (ECG-only)	56 → 90	–	91 → 80	–
Ruiz [4]	2010	Real OHCA	Compr. depth	Kalman	57.8 → 93.3	–	92.5 → 89.1	–
Irusta [8]	2009	Real OHCA	TTI	LMS	55.7 → 95.5	–	92.1 → 86.3	–
Gong [20]	2014	Porcine	Accel + TTI	Enhanced adaptive	99.3 → 93.3	–	46.8 → 96.0	–
Hajeb-M [13]	2021	Human + Real CPR	None	DNN classifier	95.2^b^	–	–	86.0^b^
Jekova [14]	2021	Real OHCA	None	End-to-end CNN	89.0^b^	–	–	91.7^b^
Li [17]	2023	Real OHCA	None	UNet + transfer	Accuracy: 65.8 → 90.8^c^			
Krasteva [15]	2023	Real OHCA	None^d^	Sliding CNN	92–94^b^	–	98–100^b^	96–97^b^
de Graaf [10]	2021	Real OHCA	TTI	cprINSIGHT	96^e^	–	98^e^	–
Didon [29]	2024	Real OHCA	None^f^	AWC	91.4–95.4^b^	–	97.5–100^b^	–
Nejad [16]^g^	2024	Human + Real CPR	None	Cascade CNNED	67.7 → 95.4	62.7 → 87.7	96.2 → 99.4	88.5 → 97.2
**Proposed** ^h^	–	Human + Real CPR	None	Cascade SBAE	72.0 → **97.5**	68.6 → **94.6**	96.6 → **99.3**	91.7 → **97.2**

TTI: transthoracic impedance; Accel: accelerometer; SE: sensitivity (%); SP: specificity (%).

a VaIues shown as “X → Y” denote unfiltered *→* after method. Ranges (“X-Y”) denote variation across experimental conditions. “–”: not reported.

b Classification-only method; value is final classifier output without separate denoising.

c Overall accuracy; SE/SP by rhythm type not separately reported.

d Requires 2–3 seconds of clean (hands-off) ECG within analysis window.

e 30% of analyses classified as indeterminate, requiring CPR pause; SE/SP exclude indeterminate cases.

f AWC operates on ECG alone; impedance traces in [29] serve only to confirm presence of compressions.

g In [16], all 52 CPR artifacts were used for both training and testing.

h 10-fold cross-validation with artifact-level separation: in each fold, the 4 test artifacts are excluded from both training and validation. Values are fold-averaged means.
